# Accuracy of Magnetic Resonance Imaging for Identifying Ovarian Cancer in a Community-Based Setting

**DOI:** 10.1089/whr.2021.0106

**Published:** 2022-01-31

**Authors:** Ruby Lin, Yun-Yi Hung, Julia Cheng, Elizabeth Suh-Burgmann

**Affiliations:** ^1^ Department of Obstetrics and Gynecology, Kaiser Permanente Northern California, San Francisco, California, USA.; ^2^ Division of Research, Kaiser Permanente Northern California, Oakland, California, USA.; ^3^ Division of Gynecologic Oncology, The Permanente Medical Group, Kaiser Permanente Northern California, Oakland, California, USA.

**Keywords:** MRI, ovarian cancer, adnexal mass, ultrasound

## Abstract

***Introduction:*** Many ovarian or adnexal masses have an indeterminate appearance on ultrasound that can raise concerns about cancer. Although magnetic resonance imaging (MRI) has been reported to reliably distinguish between benign and malignant masses, studies evaluating the accuracy of MRI in community-based practice settings are lacking.

***Methods:*** Women who underwent MRI to further evaluate an ultrasound-detected adnexal mass in 2016–2017 within a large community-based health system were identified. MRI reports were classified as favoring malignancy, benign disease, or indeterminate, blinded to pathological outcome. With a minimum of 2 years of follow-up, all ovarian cancers and borderline tumors were identified, and the accuracy of MRI assessment was determined.

***Results:*** Among 338 women who had MRI to evaluate an adnexal mass, 144 (42.6%) subsequently underwent surgery. MRI favored malignancy in 7 (4.9%) cases, benign disease in 89 (62.2%) cases, and was indeterminate in 48 (33.6%) cases. Of the seven cases in which MRI favored malignancy, two cancers and five benign tumors were found. An additional 10 cases of cancer or borderline tumor were found among women who had MRI reports that were read as indeterminate (*n* = 6) or that favored benign disease (*n* = 4). The sensitivity, specificity, positive predictive value, and negative predictive value of an MRI favoring malignancy were 16.7%, 96.2%, 28.5%, and 92.7%, respectively.

***Discussion:*** In a large community-based setting, an MRI favoring malignancy was more likely to be associated with benign disease than cancer and identified only 16.7% of true malignant cases. The findings suggest that the ability of MRI to differentiate between benign and malignant adnexal masses in community-based practice settings is currently limited.

## Introduction

Most ovarian or adnexal masses are first detected by pelvic ultrasound. However, as the ultrasound characteristics of benign and malignant masses overlap, many masses have an indeterminate appearance and can often raise concerns about ovarian cancer. Various strategies and algorithms have been suggested for differentiating between benign and malignant masses, but none have been widely adopted.^1–9^ Recently, magnetic resonance imaging (MRI) has been recommended as a means of further evaluating indeterminate adnexal masses, with studies reporting high levels of accuracy in identifying malignancy.^10–17^

However, these studies were almost exclusively done in referral-based settings in Europe in which MRI reports were read by a small number of expert radiologists, raising questions of the generalizability of the findings. Consequently, when faced with a woman with an indeterminate adnexal mass, many clinicians are uncertain whether they should obtain an MRI to further evaluate whether the mass represents ovarian cancer.

## Methods

Subjects were members of Kaiser Permanente Northern California (KPNC), a large integrated community-based health care organization that includes 19 medical centers and whose membership diversity reflects that of the surrounding community: 47% Caucasian, 7% African American, 22% Hispanic, 20% Asian or Pacific Islander, and 3% multiracial. The study was approved by the KPNC institutional review board, with a waiver of informed consent.

A standardized reporting system for ultrasound-detected adnexal masses was implemented in the health system in 2015, enabling electronic identification of women identified as having an abnormal mass on ultrasound.^18^ In brief, the system classifies adnexal masses seen on ultrasound into four risk categories (0, 1, 2, or 3) based on ultrasound characteristics: category 0 masses comprise simple cysts and are considered benign; category 1 masses are cysts with thin septation or cysts with classic benign features for a hemorrhagical cyst, dermoid tumor, endometrioma, or hydrosalpinx, and are considered probably benign; category 2 masses are primarily cystic with minor solid components (must not be >1 cm with vascular flow) and are considered indeterminate; and category 3 masses have more significant solid components or are completely solid and are considered suspicious or potentially worrisome.

The system additionally flags masses >10 cm in overall size with a designation of “X” to identify them as needing surgical consideration based on size. Patients were identified who had an abnormal adnexal mass reported on ultrasound in 2016–2017 followed by pelvic MRI within 6 months. Subjects who underwent MRI for reasons unrelated to the adnexal mass, or who had a diagnosis of ovarian cancer or metastatic cancer of any kind before MRI, were excluded.

The study period was selected as the time period when the standardized ultrasound reporting system was in place to facilitate identification of patients, while providing at least 2 years of follow-up to assess cancer outcomes. Patient age, race/ethnicity, and body mass index (BMI) at the time of the initial ultrasound were extracted from electronic databases. Within the health care setting, pelvic MRI reports are read by a group of ∼490 radiologists.

The MRI report was classified as favoring malignancy, favoring benign disease, or indeterminate by two independent reviewers (R.L. and E.S.), blinded to subsequent outcomes, based on the text of the report using the following rules: if the MRI stated that malignancy was suspected or favored or listed only malignant tumor types in a differential, the read was classified as favoring malignancy; if the report stated that benignity was suspected or favored, or listed only benign tumor types as possibilities, then the MRI read was classified as favoring benign disease; if the MRI did not indicate that either benignity or malignancy was more favored, or listed both malignant and benign tumor types in a differential, then the MRI read was considered to be indeterminate.

All surgical procedures involving the adnexa, resulting pathology, and diagnoses of ovarian or fallopian tube cancer (referred to jointly in this report as “ovarian cancers”) occurring up to December 31, 2019, were identified using electronic databases and the institution's cancer registry, confirmed by manual review. For analysis, “malignancy” was defined as either ovarian cancer or borderline tumor diagnosis.

The sensitivity, specificity, positive predictive value (PPV), and negative predictive value (NPV) of MRI for malignancy were determined when considering a “positive” MRI to be a report that favored malignancy, as well as when considering a “positive” MRI to be a report that either favored malignancy or was indeterminate. This was calculated for the cohort of women who had surgical pathology outcomes as well as for the entire cohort of women undergoing MRI, assuming benign disease for women who did not have surgery but remained clinically cancer free for at least 2 years.

All univariate, bivariate, and multivariate analyses were performed using Statistical Analysis Systems (SAS) version 9.4. Comparisons for categorical variables such as ultrasound risk score of the adnexal mass and indication for ultrasound were performed using the chi-square or Fisher's exact test. The two-sided *t*-test was used for comparing age at initial ultrasound with a *p* value of 0.05 for significance.

## Results

In 2016–2017, 338 women underwent MRI after ultrasound to further evaluate an adnexal mass. The median time interval between ultrasound and MRI was 2 weeks (interquartile range: 1–3.7 weeks). During the follow-up period (minimum of 2 years and maximum of 3 years from abnormal ultrasound), 144 (42.6%) of these women underwent surgery. Surgery was associated with larger masses, a lower BMI, and an abnormal CA 125 tumor marker level (Table 1).

**Table 1.** Characteristics of Women Who Had Magnetic Resonance Imaging to Evaluate Adnexal Mass

**Variables**	**ALL (*n*=338)**	**Surgery (*n*=144)**	**No surgery (*n*=194)**	***p***
Age (years)				0.318
Mean ± SD	46.6 ± 14.3	45.7 ± 13.5	47.2 ± 14.8	
Min–Max	18.0–85.0	19.0–83.0	18.0–85.0	
Median (IQR)	46.0 (36.0–56.0)	44.0 (36.0–54.5)	46.0 (36.0–57.0)	
Race or ethnicity				0.071
White	140 (41.4)	49 (34.0)	91 (46.9)	
Asian/Pacific Islander	76 (22.5)	38 (26.4)	38 (19.6)	
Hispanic	72 (21.3)	38 (26.4)	34 (17.5)	
Black	28 (8.3)	10 (6.9)	18 (9.3)	
Native American/multiracial/unknown	22 (6.5)	9 (6.3)	13 (6.7)	
BMI (kg/m^2^)				0.006
≤30	230 (68.0)	86 (59.7)	144 (74.2)	
31–35	42 (12.4)	20 (13.9)	22 (11.3)	
>35	50 (14.8)	32 (22.2)	18 (9.3)	
Missing	16 (4.7)	6 (4.2)	10 (5.2)	
Size of mass on initial ultrasound (cm)				<0.0001
Mean ± SD	6.1 ± 4.0	7.3 ± 3.8	5.1 ± 3.9	
Min–Max	1.0–27.0	1.0–21.0	1.0–27.0	
Median (IQR)	5.0 (3.0–8.0)	6.0 (4.0–10.0)	4.0 (3.0–6.0)	
CA 125				<0.0001
Abnormal	26 (7.7)	18 (12.5)	8 (4.1)	
Missing	203 (60.1)	67 (46.5)	136 (70.1)	
Normal	109 (32.2)	59 (41.0)	50 (25.8)	

BMI, body mass index; IQR, interquartile range; SD, standard deviation.

**FIG. 1.**
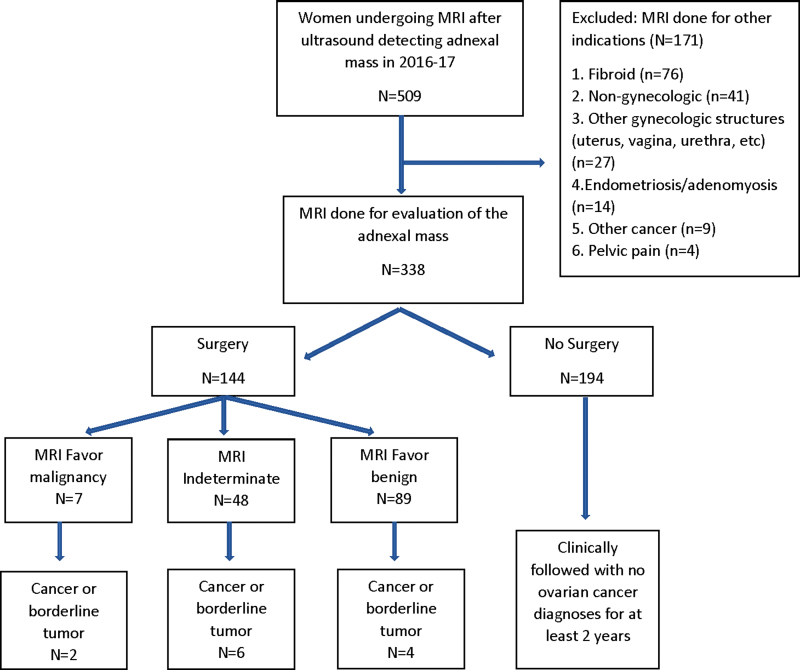
Flowchart of cohort assembly. MRI, magnetic resonance imaging.

Among the 144 women who had surgery, MRI favored malignancy in 7 (4.9%), benign disease in 89 (62.2%), and was indeterminate in 48 (33.6%) women. Ovarian cancer was found at surgery in 11 (7.7%) patients, borderline tumor in 1 (0.7%) patient, and benign disease in 131 (91.6%) patients. No additional cases of ovarian cancer were diagnosed outside of surgery. Of the 12 patients found to have a cancer or borderline tumor, MRI favored malignancy in 2 cases (16.7%), benign disease in 4 cases (33.3%), and was indeterminate in 6 cases (50%). Conversely, for the seven patients with an MRI favoring malignancy, cancer was found in two (28.6%) patients and benign disease in five (71.4%) patients. A flowchart of MRI results and outcomes is shown in Figure 1.

When the MRI interpretation was compared with the standardized ultrasound report for the two cases wherein MRI correctly identified malignancy, the ultrasound was read as suspicious in one case and probably benign in one case. In the latter case, the ultrasound was read as probable hydrosalpinx and the patient was found to have a fallopian tube cancer. Of the nine additional cases of cancer in whom MRI was indeterminate or favored benign disease, the ultrasound was read as suspicious in eight cases and indeterminate in one case (Table 2).

**Table 2.** Comparison of Ultrasound and Magnetic Resonance Imaging Interpretation in Malignant Cases

**Case**	**Pathology**	**Ultrasound read**	**MRI read**
1	Serous ovarian	Cystic with major solid vascular component or solid (suspicious)	Indeterminate
2	Serous ovarian	Cystic with major solid vascular component or solid (suspicious)	Indeterminate
3	Serous tubal	Hydrosalpinx/probably benign	Malignant
4	Endometrioid	Cystic with major solid vascular component or solid (suspicious)	Benign
5	Endometrioid	Cystic with major solid vascular component or solid (suspicious)	Malignant
6	Mixed endometrioid/clear cell	Large (>10 cm) cystic with major solid vascular component or solid (suspicious)	Indeterminate
7	Carcinoid	Large (>10 cm) cystic with major solid vascular component or solid (suspicious)	Benign
8	Granulosa cell	Cystic with major solid vascular component or solid (suspicious)	Indeterminate
9	Granulosa cell	Cystic with minor solid component (indeterminate)	Indeterminate
10	Granulosa cell	Cystic with major solid vascular component or solid (suspicious)	Benign
11	Dysgerminoma	Large (>10 cm) cystic/solid or solid (suspicious)	Indeterminate
12	Endometrioid borderline	Probable hemorrhagical cyst (probably benign)	Indeterminate

MRI, magnetic resonance imaging.

Table 3 gives the sensitivity, specificity, PPV, and NPV of a “positive” MRI, if only MRI reports favoring malignancy are considered positive for cancer, as well as if indeterminate reports are also considered positive for cancer. MRI had a sensitivity of 16.7%, meaning MRI favored malignancy in only 16.7% of malignant cases. The PPV of MRI, or the probability that a woman with an MRI favoring malignancy would be found to have malignancy, was 28.5%. Similar results were obtained when the calculations were performed for the entire cohort, assuming benign disease for women who did not have surgery but remained cancer free for at least 2 years.

**Table 3.** Sensitivity, Specificity, Positive Predictive Value and Negative Predictive Value of Magnetic Resonance Imaging for Malignancy

	**Among women who had surgery (*n* = 144)**	**Among all women having MRI (*n* = 338)^a^**
If MRI reports that favored malignancy are considered positive for cancer		
Sensitivity, %	16.7 (2.1–48.1)	16.7 (2.1–48.1)
Specificity, %	96.2 (91.4–98.8)	98.5 (96.5–99.5)
Positive predictive value, %	28.5 (7.9–64.8)	28.9 (8.0–65.3)
Negative predictive value, %	92.7 (90.8–94.3)	96.9 (96.1–97.6)
If MRI reports that favored malignancy or were indeterminate are considered positive for cancer		
Sensitivity, %	66.7 (34.9–90.1)	66.7 (34.9–90.1)
Specificity, %	64.4 (55.6–72.5)	74.9 (69.8–79.5)
Positive predictive value, %	14.5 (9.7–21.2)	9.0 (6.0–13.3)
Negative predictive value, %	95.5 (90.5–98.0)	98.4 (96.4–99.3)

^a^ Assumes benign disease for women who did not have surgery but remained clinically cancer free for at least 2 years after MRI.

## Discussion

In a large community-based setting in which MRI reports are read by a diverse group of radiologists, MRI did not accurately differentiate between benign and malignant adnexal masses. MRI favored malignancy in only 16.7% of malignant cases, and an MRI report favoring malignancy was more likely to be associated with benign disease.

In our study, the overall prevalence of ovarian cancer in the population was low (12 cancers or borderline tumors among 338 women = 3.5%), which is the primary reason the NPV is high despite the relatively poor sensitivity and specificity. If only MRI reports that clearly favor malignancy are considered positive tests, the PPV was 28.5% and the NPV was 92.7%. The question of whether repeated MRI reports would improve accuracy is not addressed by this study but, in general, repeating tests for the same person reduces the error rate to the extent that the error is random (the likelihood of error is independent across consecutive tests).

Although it is possible there is some element of random error in reading MRI, perhaps related to the reader, it seems likely that there are also specific patient or tumor factors that affect the performance of MRI. Three of the cancers were granulosa cell tumors that tend to be more solid. In these cases, MRI was read as indeterminate in two cases and favored benign in one case, suggesting that the differentiation between benign and malignant masses by MRI may be particularly challenging for predominantly solid tumors.

The only case in which MRI performed better than ultrasound in identifying a cancer was that of a mass read as a large hydrosalpinx on ultrasound that was found to be a fallopian tube cancer. It is possible that there may be specific types of masses such as large hydrosalpinges that are difficult to fully characterize by ultrasound where MRI may provide an advantage. In any case, the main value of repeated tests arises from the comparison of the mass with itself over time, since stability argues strongly against malignancy regardless of imaging modality.

To our knowledge, this is the first report to describe the accuracy of MRI for differentiating benign from malignant adnexal masses in a U.S. community-based practice setting. In a systematic review of the literature, six studies that evaluated the ability of MRI to differentiate between benign and malignant adnexal masses were identified, all of which were noted in the review to be subject to substantial potential bias.^10^ In all the studies cited, MRI reports were interpreted by one or two expert readers to identify cancer in a tertiary care setting.^11–17^ Our findings raise questions about how generalizable these results are to community settings in the United States. The degree to which the discrepancy is attributable to differences in expertise versus different systems of reporting is unclear.

An interesting finding was that lower BMI was associated with surgical intervention. The reasons for this are unclear but suggest that perceived surgical risk played a role in determining clinical management.

The main limitation of this study is that in this and nearly all practice settings, MRI interpretations are reported using an unstructured narrative format, which necessitated that the reports be secondarily classified in terms of whether malignancy or benign disease was favored. Although this was done by two independent reviewers blinded to outcome, it is possible that some reports, particularly those classified as indeterminate, were not intended to be interpreted in that way by the reading radiologist. However, even when indeterminate MRI reports were counted as being “positive” for cancer, the sensitivity remained relatively poor at 66.7%, whereas the PPV worsened to only 14.5%.

Furthermore, the need for clinicians to “interpret” MRI reports reflects what occurs in current clinical practice, since the narrative unstructured format of MRI reports results in considerable variability in reporting styles and terminology. It is possible that performance would improve if readers were required to assess and report malignancy risk using a standardized lexicon and scoring system in which readers were required to assess and report malignancy risk according to a standardized system.^19^

In addition, since MRI is not routinely obtained after ultrasound, there was undoubtedly selection bias for who underwent MRI toward women for whom there was some additional concern driving further evaluation. However, selection bias toward increased cancer risk among women who get MRI is appropriate from a clinical standpoint and would be expected to enhance, rather than hinder, the performance of MRI for detecting cancer.

Study strengths include the diverse community-based nature of the population studied, which increases generalizability to other community-based practice settings. The closed integrated nature of the health system minimizes the likelihood that surgeries or cancer diagnoses were undetected. In addition, cancer diagnoses made at other institutions within California would have been captured by the institution's participation in the California Cancer Registry. Finally, unlike previous studies, we assessed the performance of MRI not only for women who underwent surgery but for the entire cohort of women undergoing MRI as well, since the PPV and NPV of MRI are most relevant to women when determining whether surgery is necessary.

## Conclusion

In summary, we found that MRI did not accurately differentiate between benign and malignant adnexal masses in a large community-based setting. Further research is needed to understand the factors affecting MRI performance and whether a standardized lexicon and risk stratification scoring system can improve performance in community-based settings.

## Abbreviations Used

BMI: body mass index
IQR: interquartile range
KPNC: Kaiser Permanente Northern California
MRI: magnetic resonance imaging
NPV: negative predictive value
PPV: positive predictive value
SAS: Statistical Analysis Systems
SD: standard deviation
